# Government environmental regulation and farmers' engagement in traditional folk practices: the mediating roles of ecological cognition and social norms

**DOI:** 10.3389/fpsyg.2025.1704337

**Published:** 2026-01-12

**Authors:** Qing Wu, Jiaxiao Feng, Xiaoshi Liu, Yanli Huang

**Affiliations:** 1 Tourism and Historical Culture College, Zhao Qing University, Zhaoqing, China; 2 Faculty of International Tourism and Management, City University of Macau, Taipa, Macao SAR, China; 3 College of Urban and Environmental Sciences, Central China Normal University, Wuhan, China; 4 School of Economics and Management of Zhaoqing University, Zhaoqing, China

**Keywords:** ecological civilization, ecological cognition, environmental regulation, rural cultural governance, social norms

## Abstract

**Introduction:**

As an important carrier of cultural identity and community cohesion in rural Chinese society, traditional folk activities often generate tensions with modern ecological protection goals due to their resource-intensive characteristics. This study takes the millennium-old “Firecracker Lion Dance” activity in Deqing County, Guangdong Province, as a research case to explore how government environmental regulation influences farmers' willingness to participate in the Sustainable Firecracker-Lion Dance Custom, with a particular focus on the mediating roles of social norms and ecological cognition.

**Methods:**

Based on 423 valid samples collected through fieldwork from 2022 to 2024, a structural equation model was constructed to systematically examine the differential regulatory pathways of incentive-based and coercive environmental regulation on farmers' participation in traditional folk practices, as well as the mediating roles of ecological cognition and social norms.

**Results:**

The results show that incentive-based regulation significantly enhances farmers' willingness to participate through motivational mechanisms (β = 0.229, *p* < 0.001), whereas coercive regulation shows no significant effect. Social norms exert significant positive effects on participation willingness (descriptive norms: β = 0.167, *p* < 0.001; injunctive norms: β = 0.238, *p* < 0.001), reflecting the behavioral constraints of group identity and opinion orientation in rural acquaintance societies. Meanwhile, ecological cognition significantly inhibits participation willingness (β = −0.210, *p* < 0.001) and exhibits a negative mediating effect in the coercive regulation path.

**Conclusion:**

This study provides deeper insight into that the effectiveness of environmental regulation in traditional folk practices depends less on regulatory intensity than on cultural compatibility. Social norms function as key cultural conduits that translate policy signals into behavioral acceptance, whereas ecological cognition may generate value tensions that constrain participation. The results of this study offer theoretical validation and practical significance for the integrated governance of culture and ecology within the paradigm of ecological civilization.

## Introduction

In the context of global ecological governance, striking a balance between environmental sustainability and cultural heritage preservation has emerged as a pressing concern (Aboulnaga et al., 2024; Holtorf, 2013). The Chinese government's concept of “ecological civilization” emphasizes the integrated advancement of environmental protection and socio-cultural practices. However, national-level environmental regulations often face substantial resistance during implementation in rural areas with deeply rooted traditional folk customs. Take Deqing County in Guangdong Province as an example. The “Lion Dance to Celebrate the New Year” is a widespread custom during the Spring Festival that has been passed down for over a thousand years. This ritual symbolizes not only the ushering in of good fortune and the departure of the old year but also the fostering of strong community cohesion and cultural identity. During performances, villagers show their respect and festive joy by igniting fireworks and firecrackers aimed at the lion dancers. As living standards have risen and symbolic meanings have deepened, the event has grown in scale, accompanied by increasingly intensive firecracker use. However, the resulting air pollution and noise issues have gradually brought this tradition under the scrutiny of environmental policy. While regulations are intended to reduce ecological stress, top-down enforcement mechanisms can conflict with local cultural traditions and may even weaken villagers' emotional connections to their folk practices (Adger et al., 2013). Recently, governments increasingly guide their transformation toward greener, more sustainable forms, retaining symbolic values while reducing ecological harm. Hence, investigating how environmental regulations influence farmers' participation in traditional customs not only contributes to enhancing the cultural adaptability of environmental policies but also offers practical insights for the sustainable inheritance of folk traditions. Existing studies have primarily concentrated on the direct effects of environmental regulations on individual pro-environmental behaviors (Stern, 2000). However, the mediating mechanisms that link these regulations to cultural practices, such as the interplay between social norms and ecological cognition, have not been thoroughly examined. In collective rituals like the firecracker “attacks” on lion dancers, social norms exert a powerful influence on individual behavior. The informal rules embedded in local culture guide villagers' actions through group pressure (Gelfand et al., 2020). On the one hand, social norms can both reinforce adherence to customs and undermine the effectiveness of policies. on the other, as environmental risks become more apparent, individuals' ecological cognition may significantly influence their participation behavior. To explore this dynamic, this study develops a structural equation model that integrates environmental regulation, social norms, and ecological cognition to examine farmers' willingness to engage in the Sustainable Firecracker–Lion Dance Custom (S-FLC). The findings aim to offer theoretical insights for culturally adaptive environmental governance and practical guidance for the sustainable preservation of folk traditions.

## Literature review and hypothesis

### Environmental regulation and farmers' willingness to participate in S-FLC

Environmental regulation is widely acknowledged as an essential policy tool for government intervention in ecological governance, aiming to address market failures and reduce environmental externalities (Du et al., 2024). Its primary objective is to promote the sustainable use of public resources and ensure the stable provision of ecosystem services through institutional arrangements (Li M. et al., 2022; Stern, 2000). As global challenges, such as climate change and regional ecological degradation, intensify, environmental regulation has shifted from traditional command-and-control models to more flexible, incentive-based, and market-oriented tools (Tietenberg, 1998).

In China, the recent implementation of market-based instruments, including emissions trading (Li et al., 2024), ecological compensation (Cao et al., 2022; Ren et al., 2023), and green fiscal policies has injected new momentum into environmental governance through institutional innovation. Academic discussions have consistently examined the relationship between the stringency of environmental regulations and their governance outcomes (Zhao et al., 2022). For example, (Cai and Guo, 2023) employed fixed-effects and threshold models to reveal an inverted U-shaped relationship between the intensity of regulation and the effectiveness of pollution control. Specifically, low levels of regulatory pressure fail to exert meaningful constraints, whereas effectiveness improves significantly once a certain threshold is reached. This finding highlights that the efficacy of environmental regulation depends not only on institutional design but also on the coordination between regulatory intensity and enforcement capacity (Lu et al., 2022). However, most existing research has concentrated on the impact of environmental regulation within industrial systems or urban ecosystems (Dai et al., 2023; Liu et al., 2024), with relatively limited attention paid to the complex interactions between environmental regulation and traditional cultural practices in rural areas. Rural ecological spaces are often hybrid landscapes shaped by both natural and cultural elements. Folk customs not only preserve cultural memory and foster social cohesion but are also intricately linked to local patterns of ecological resource use.

With the ritualization, commercialization, and scaling up of folk practices, their environmental impact has intensified, leading to increasing tensions between cultural continuity and ecological conservation (Aptasari et al., 2024; Gibson, 1993). In Deqing County, the firecracker-lion dance ritual boasts a history of over a thousand years. However, in recent years, the rising volume of fireworks has raised significant environmental concerns. Research by Nishanth revealed that continuous firework use during the Vishu festival in India resulted in a doubling of ozone and PM10 concentrations, while nitrogen dioxide levels increased by 2.5 times (Nishanth et al., 2012). These pollution issues pose new challenges for regulatory frameworks. Therefore, the implementation of environmental regulatory policies faces significant cultural constraints and challenges related to social negotiation (Guo and Schneider, 2015). Rural residents often regard participation in traditional folk customs as essential for expressing group belonging, maintaining interpersonal relationships, and preserving local identity. However, public awareness of ecological protection and pollution control is still relatively limited, resulting in tension between environmental regulations and established cultural practices. In these situations, environmental policy design must extend beyond mere technical feasibility and economic incentives; it must also consider the cultural context and the social-psychological foundations of local communities. Specifically, environmental regulation can be broadly categorized into two types: incentive and coercive (Hu et al., 2020; Tang et al., 2022). The former focuses on encouraging villagers to adopt more environmentally friendly forms of folk practice through tools such as financial subsidies, honorary incentives, and green certifications. For instance, they could replace polluting activities with low-smoke, eco-friendly firecrackers or implement symbolic, non-material rituals like “symbolic vegetable plucking.” The latter emphasizes strict control through legal mandates, administrative orders, and prohibition zones—establishing environmental red lines and banning high-pollution behaviors altogether. In practice, these two regulatory paths are often implemented in tandem, forming a combined framework of “soft-hard” policy integration. Environmental regulation does not aim to directly restrict farmers' cultural expressions but rather to steer the firecracker–lion dance toward greener and more sustainable forms while preserving its symbolic and social values. Consequently, the willingness to participate discussed in this study pertains to community members' readiness to engage in these Sustainable Firecracker–Lion Dance Customs (S-FLC). Based on this analysis, this study proposes the following hypothesis:

H1: Environmental regulation has a positive effect on farmers' willingness to participate in the S-FLC.

H1a: Incentive-based environmental regulation positively influences farmers' willingness to participate in the S-FLC.

H1b: Coercive environmental regulation positively influences farmers' willingness to participate in the S-FLC.

### Social norms and farmers' willingness to participate in S-FLC

Social norms are informal behavioral rules that develop gradually among members of a social group through continuous interaction (Dannals and Li, 2024; Zhang et al., 2023). These norms are rooted in shared expectations and social evaluation mechanisms within the group, exerting a lasting influence on individual behavior (Mueller and Sieck, 2009). As a key means of social control, social norms serve not only as components of informal institutions but also as a collective behavioral regulatory mechanism embedded within social structures and cultural traditions (Van Kleef, 2024). Through implicit mechanisms of reward, punishment, and a sense of belonging, social norms align individual behavior with group expectations, thereby maintaining social order and group stability (Legros and Cislaghi, 2020). Recently, scholars have increasingly focused on the role of social norms in influencing environmental behaviors (Akçay and Cleve, 2021). The underlying logic lies in the establishment of behavioral references, specifically “what others are doing” (descriptive norms) and “what others believe I should do” (injunctive norms), which can effectively motivate individuals to adopt pro-environmental behaviors. In an experimental study on household energy use, Schultz et al. (2018) found that participants who were informed that most of their neighbors conserved electricity significantly reduced their own energy consumption. Moreover, when such information was paired with a moral obligation prompt (i.e., an injunctive norm), the energy-saving behavior was more sustained over time (Schultz et al., 2018). This suggests that both descriptive norms, based on observed behaviors of others, and injunctive norms, grounded in social value judgments, can shape individuals' cognition and emotions, thereby exerting normative influence on behavior. Niu et al. (2023) further extended the understanding of social norms in high-cost behavioral contexts. Their research demonstrated that even when environmental behaviors incur considerable personal costs (e.g., financial or time-related), social norms can still significantly enhance individuals‘ willingness to act through activating internal motivations and a desire for group belonging. Under certain conditions, social norms may also serve a mediating role in influencing behavior (Niu et al., 2023).

Although social norms have been extensively theorized and empirically examined in the field of environmental behavior (Bai and Bai, 2020; Niu et al., 2023; Perry et al., 2021), their role in the context of traditional folk activities remains relatively underexplored. In particular, there remains a lack of systematic explanation about how social norms affect individuals' behavioral choices when participating in cultural ritual-based activities. As a deeply rooted and highly ritualized local custom, the firecracker-lion dance not only carries symbolic meaning for the village community but also serves as a crucial vehicle for villagers' identity construction and cultural memory. In such events, individual participation decisions are rarely driven purely by personal preference; instead, they are frequently influenced by collective expectations, existing social customs, and comparisons with peers. In particular, descriptive social norms affect behavioral tendencies by demonstrating what is considered “typical behavior” within the group. When most households in a village decide to set off firecrackers during the Spring Festival as part of the lion dance ritual, other farmers may choose to participate as well to avoid feeling like outliers or being seen as socially deviant. Injunctive social norms, on the other hand, reflect value judgments and provide moral guidance regarding specific behaviors. Engaging in the firecracker-lion dance may be framed as an act of “respecting tradition” or “preserving cultural heritage.” Such moral narratives, reinforced through public opinion and social evaluation, further amplify conformity behavior among farmers.

Moreover, social norms may function as mediating or moderating mechanisms in the effectiveness of government-led environmental regulations (Hua and Mi, 2023). On one hand, when a majority of individuals recognize and adhere to environmental norms, social norms can act as “contextual catalysts” that enhance the behavioral effectiveness of the policy (Yamin et al., 2019). On the other hand, if the dominant community persists in high-pollution traditional practices, social norms may weaken the policy's guiding influence and contribute to collective behaviors characterized as “irrational resistance” (Chen Q. et al., 2023). Therefore, as traditional customs transition toward more sustainable practices, social norms play a crucial role in influencing farmers' willingness to participate in the Sustainable Firecracker–Lion Dance Custom (S-FLC). Based on the literature and theoretical rationale presented, this study proposes the following hypothesis:

H2: Social norms have a positive effect on farmers' willingness to participate in the S-FLC.

H2a: Descriptive social norms positively influence farmers' willingness to participate in the S-FLC.

H2b: Injunctive social norms positively influence farmers' willingness to participate in the S-FLC.

H3: Social norms mediate the relationship between environmental regulation and farmers' willingness to participate in the S-FLC.

H3a: Descriptive social norms mediate the relationship between incentive-based environmental regulation and farmers' willingness to participate in the S-FLC.

H3b: Descriptive social norms mediate the relationship between coercive environmental regulation and farmers' willingness to participate in the S-FLC.

H3c: Injunctive social norms mediate the relationship between incentive-based environmental regulation and farmers' willingness to participate in the S-FLC.

H3d: Injunctive social norms mediate the relationship between coercive environmental regulation and farmers' willingness to participate in the S-FLC.

### Ecological cognition and farmers' willingness to participate in S-FLC

Ecological cognition refers to the knowledge system that individuals gradually construct through continuous information acquisition and practical experience, encompassing understanding of the natural environment, ecosystem functioning, and the impacts of human behavior (Piekarski et al., 2016). This concept typically includes three dimensions: first, the ability to identify environmental problems. Second, the ability to understand causal relationships; and third, the ability to explore feasible solution (Kollmuss and Agyeman, 2002). Ecological cognition is not merely a static stock of knowledge but a dynamic cognitive capacity that is embedded within one's value systems, risk perceptions, and behavioral decision-making processes. Research has shown that ecological cognition plays a crucial role in explaining and predicting pro-environmental behaviors. For example, (Meng and Si, 2022) found that farmers' awareness of ecological value significantly enhanced their tendency to engage in environmentally friendly behaviors, both directly and indirectly by improving their environmental attitudes.

Currently, ecological cognition is widely applied in research related to agricultural ecological transformation, green consumption, and waste recycling. Ren et al. (2022) utilizing a “dual barriers model,” found that although ecological cognition increases farmers' willingness to adopt green production practices, its direct impact on behavior is limited by constraints such as available livelihood resources. Chen et al. (2022) further noted that ecological cognition not only enhances consumers' willingness to engage in beverage packaging recycling but also has a more pronounced effect when supported by descriptive social norms. However, compared with studies on green production or urban residents' behavior, research on the role of ecological cognition in decision-making regarding traditional folk customs remains preliminary. Folk customs, which are deeply embedded in local identity and cultural heritage, are often influenced by family values, religious beliefs, and group expectations, rather than solely by rational evaluation (Gonçalves and Gonçalves, 2022). Therefore, understanding the role of ecological cognition requires situating it within a multi-dimensional mechanism linking social culture, cognition, and behavior.

Theoretically, individuals with higher ecological cognition tend to exhibit greater environmental sensitivity and awareness of ecological risks, making them more likely to avoid behaviors that are highly polluting or environmentally costly (Ma et al., 2021). For example, the firecracker-lion dance generates a strong festive atmosphere and holds symbolic meaning; however, the large-scale use of firecrackers results in significant air and noise pollution. Even though the firecracker-lion dance has undergone safety and environmental improvements under policy guidance, participants with high ecological awareness may still perceive residual noise and air pollution, which may lead them to engage more cautiously or selectively (Lewis and Dowsey-Magog, 1993). As the level of ecological cognition increases, individuals tend to assess pollution consequences more rationally and with a forward-looking perspective, resulting in a lower tendency to participate (Nie et al., 2020).

Moreover, ecological cognition may serve as a bridging mechanism between government environmental regulation and farmers' willingness to participate in S-FLC. On the one hand, a higher level of ecological cognition can enhance individuals' understanding of regulatory goals and recognition of policy legitimacy, thereby increasing their willingness to comply with regulations (Chen W. et al., 2023). On the other hand, improved ecological cognition may encourage individuals to internalize policy objectives as personal behavioral guidelines, leading to a shift from passive acceptance to active self-adjustment (Nie et al., 2020). Consequently, ecological cognition could strengthen environmental regulation by deepening farmers' understanding of regulatory goals and reinforcing their recognition of policy legitimacy, which promotes the internalization of policy intentions and shifts behavior from passive compliance to proactive sustainability support. Based on this analysis, the following hypotheses are proposed:

H4: Ecological cognition negative affects farmers' willingness to participate in the S-FLC.

H5: Ecological cognition mediates the relationship between environmental regulation and farmers' willingness to participate in the S-FLC.

H5a: Ecological cognition mediates the relationship between incentive-based environmental regulation and farmers' willingness to participate in the S-FLC.

H5b: Ecological cognition mediates the relationship between coercive environmental regulation and farmers' willingness to participate in the S-FLC.

**Figure 1:**
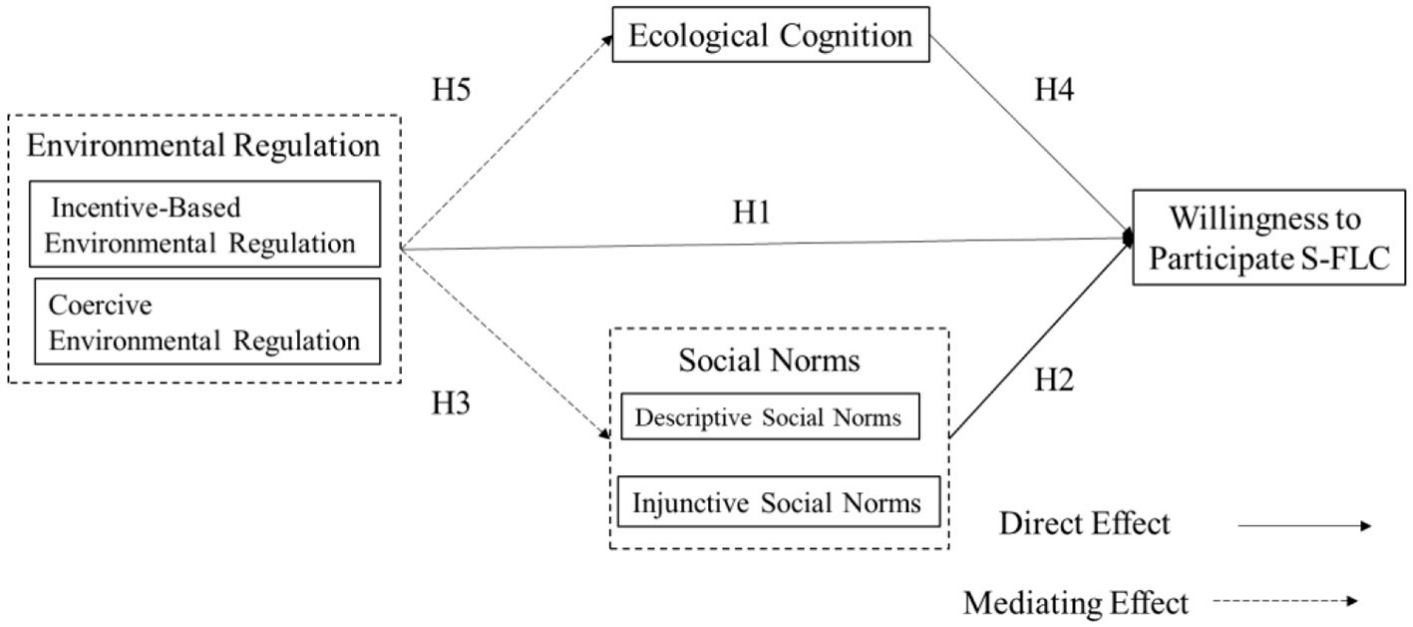
Theoretical framework of the effects of environmental regulation, social norms, and ecological cognition on farmers' S-FLC participation.

Through this analysis, the study develops a theoretical framework that combines environmental regulation, social norms, and ecological cognition to investigate their impact on farmers' willingness to engage in S-FLC (see Figure 1).

## Case description and data sources

### Case overview

The custom of “Firecracker Attacks on the Lion”, locally referred to as “Lion Bombing”, is a unique ritual celebrated during the Spring Festival and Lantern Festival in Deqing County, located in Zhaoqing City, Guangdong Province. Historical records indicate that this tradition dates back to the Song Dynasty, boasting a history of over 1,000 years. Throughout the festive season, Deqing is adorned with vibrant lanterns, and the air is filled with the sounds of firecrackers. Farmers gather around the lion dance teams, carrying boxes of firecrackers. They burn incense sticks to ignite continuous strings of firecrackers, which are detonated from front to back in a symbolic act of “attacking” the lion—an expression of good wishes for the new year, expressing hopes for favorable weather and a bountiful harvest. The lion dance is performed by two dancers, one controls the lion's head while the other manages the tail, accompanied by a figure dressed as the “Laughing Buddha” and the rhythm of traditional drums.

**Figure 2:**
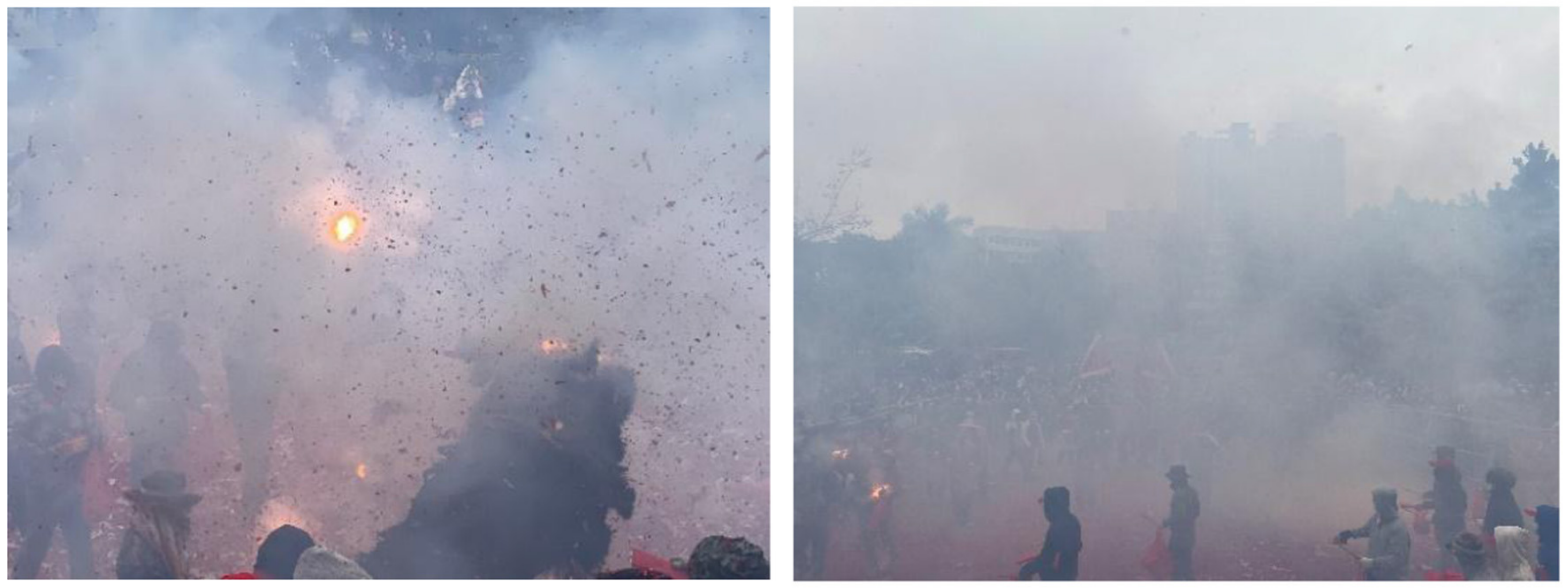
On-site scene of the firecracker-lion dance folk activity in Deqing County.

Dancers wear helmets, fire-resistant suits, and cloth arm wraps to protect themselves from firecracker debris. Despite the deafening explosions and thick smoke, they must maintain the rhythm and posture of the lion dance, demonstrating exceptional physical coordination and psychological resilience (see Figure 2). This ritual not only embodies the rich festive atmosphere and collective ritual sense characteristic of southern Guangdong but also serves as a vital medium through which local residents reinforce cultural identity and social cohesion. It has become one of the most vibrant and eagerly anticipated celebrations in the region. However, with the expansion of the event's scale, growing environmental concerns have emerged, particularly related to the high-intensity noise and airborne particulate matter generated by massive firecracker use. On one hand, the activity causes severe air pollution. Nishanth et al. (2012) found that firework emissions during festivals can double ozone (O3) concentrations and sharply increase nitrogen oxides (NO_2_, NO3), posing significant health risks to vulnerable groups such as children, the elderly, and individuals with respiratory conditions. On the other hand, noise pollution is especially prominent. The simultaneous explosion of firecrackers and roaring lion drums can produce sound levels exceeding 100 decibels—far surpassing the nighttime environmental noise limit of 55 dB(A)—which significantly disturbs nearby residents. Beyond disrupting daily life, such noise pollution also raises public health and social order concerns. Recently, environmental issues have become a focal point of public discourse (Münzel et al., 2024; Perry et al., 2021). In this background, the local government has implemented safety-oriented environmental regulations, including mandatory approval procedures, on-site risk assessments, standardized firecracker management, temporary traffic control, and public safety education. These measures aim to guide the custom toward a safer and greener form. This development has given rise to a new environmental governance dilemma in which cultural identity and ecological sustainability are in tension.

### Data sources

The data used in this study were collected through a three-year systematic field investigation conducted by the research team in Deqing County, Guangdong Province, from January 2022 to February 2024. A stratified random sampling method was employed, with Deqing's county seat as the geographic center, extending to surrounding major towns and administrative villages. Sample site selection considered geographic distance, population density, and the level of activity in folk customs to ensure both regional representativeness and diversity. During fieldwork, the research team adopted a mixed-methods approach that combined structured questionnaire surveys with in-depth interviews. The questionnaire survey primarily targeted local farming households. The content covered multiple dimensions: respondents' basic demographic information; their perceptions and experiences of government-led environmental regulation, both incentive-based and coercive types; their acceptance of and compliance with descriptive and injunctive social norms; their level of awareness of ecological and environmental issues (i.e., ecological cognition); and their willingness and behavioral tendencies to participate in Sustainable Firecracker-Lion Dance Custom (S-FLC).

All questionnaires were administered using a “face-to-face household distribution—with on-site explanation and immediate collection” procedure by trained enumerators to maximize response accuracy and data reliability. Prior to the official survey, several rounds of preparatory work and training sessions were held to ensure the data's accuracy and reliability. These included logical consistency testing of the questionnaire, standardization of terminology explanations, and training in interview techniques and research ethics. All team members involved had academic backgrounds in related disciplines and were experienced in rural fieldwork. In total, 566 paper-based questionnaires were distributed, and 423 valid responses were collected, resulting in a valid response rate of 74.73%. The basic characteristics of the sample are summarized in Table 1. The majority of respondents were local residents, comprising 83.0% of the total sample, while tourists made up 17%. In terms of gender distribution, a larger proportion of respondents were female. The sample was predominantly composed of young and middle-aged adults, with individuals aged 18–40 accounting for 78.0%. Regarding length of residence, 47.0% of respondents had lived in the area for more than 20 years, while 18.4% had resided there for 5 years or less. Concerning education level, over 50% of respondents held at least a college diploma or a bachelor's degree.

**Table 1:** Basic characteristics of survey respondents.

**Category**	**Item**	**Frequency (n)**	**Percentage (%)**
Residence status	Local resident	351	83.0%
	Tourist	72	17%
Gender	Male	165	39.0%
	Female	258	61.0%
Age(years old)	18–25	173	40.9%
	26–30	60	14.2%
	31–40	97	22.9%
	41–50	58	13.7%
	51–65	35	8.3%
Length of residence (year)	≤ 5 years	78	18.4%
	6–10 years	30	7.2%
	11–15 years	39	9.2%
	16–20 years	77	18.2%
	>20 years	199	47.0%
Education level	Junior high school or below	48	11.3%
	High school or vocational	135	32.0%
	College diploma or bachelor's degree	237	56.0%
	Postgraduate or above	3	0.7%

## Variable description

To thoroughly investigate how environmental regulation affects farmers' willingness to engage in traditional folk customs, this study analyzes the mediating roles of ecological cognition and social norms. Drawing on previous research and empirical findings from fieldwork, we applied refined measurement designs to each variable to enhance conceptual clarity and empirical rigor.

### Dependent variable: willingness to participate in folk customs

In this study, the dependent variable refers to farmers' willingness to participate in Sustainable Firecracker-Lion Dance Custom (S-FLC). The measurement is adapted from the cultural tourism participation scale proposed by Kim and Seock (2019), with contextual modifications based on the local characteristics of Deqing's folk customs. Specifically, 5 measurement dimensions are included: (1) the respondent's basic knowledge of the firecracker-lion dance ritual; (2) personal preference for the activity; (3) attitude toward supporting its continued performance; (4) willingness to actively participate in the activity; and (5) intention to participate in the custom within the next year. All items are measured using a five-point Likert scale, where 1 indicates “strongly disagree/unwilling,” and 5 indicates “strongly agree/very willing.”

### Independent variable: environmental regulation

Environmental regulation is established as the core independent variable and is categorized into two dimensions: incentive-based and coercive regulation. Incentive-based regulation influences farmers' behavior primarily through soft measures, such as education and incentives, while coercive regulation depends on rigid mechanisms, including legislation and administrative penalties. The measurement tool is adapted from Stern's model of policy acceptance and regulatory response (Stern, 2000), with contextual adjustments based on the fieldwork conducted for this study. Four measurement items are included: (1) the respondent's awareness of government publicity and educational efforts regarding firecracker use; (2) understanding of the government's restrictive policies on firecracker ignition; (3) attitudes toward governmental penalties or criticism for illegal firecracker use; and (4) agreement with the idea of rewarding or recognizing individuals who comply with firecracker bans. Collectively, these items represent farmers' cognitive and attitudinal responses to regulatory measures.

### Mediating variables: ecological cognition and social norms

This study introduces ecological cognition and social norms as potential mediating variables to explore their roles in the behavioral pathways linking environmental regulation to participation in folk customs.

(1) Ecological Cognition: this construct measures farmers' awareness and understanding of environmental issues, focusing on two key aspects: (i) the extent to which they agree on the significance of environmental protection and (ii) their perception of risks related to environmental problems caused by fireworks, including air and noise pollution. The design of the scale is based on the Environmental Attitudes and Intelligence Scale (EAI) created by Milfont and Duckitt (2010), with contextual modifications tailored to field conditions.

(2) Social Norms: social norms are divided into two dimensions: descriptive norms and injunctive norms, corresponding respectively to “prevalent behavioral trends” and “social expectations to be followed.” The measurement draws on the normative influence models proposed by Rimal and Real (2005) and Sparkman and Walton (2017), including (i) the perceived frequency with which neighbors participate in the firecracker-lion dance (descriptive norms) and (ii) social evaluation and moral attitudes regarding whether one ought to participate in the activity (injunctive norms). This dual-dimensional approach provides a more comprehensive understanding of how group norms regulate individual behavior.

### Control variables

To eliminate the potential confounding effects of individual characteristics on the dependent variable, the study incorporates five control variables: (1) household identity (e.g., head of household, spouse, other family member); (2) gender; (3) age; (4) length of residence in the village (measured in years); and (5) education level.

## Model reliability and validity testing

### Reliability and validity assessment

To ensure the stability and accuracy of scale measurements in the empirical study, this paper conducted a systematic reliability and validity analysis using SPSS 24.0 and AMOS 26.0, based on the collected questionnaire data. First, from the perspective of reliability, Cronbach's alpha coefficient was employed to assess the internal consistency of each latent variable. The results indicate that the overall scale achieved a Cronbach's alpha value of 0.830, which exceeds the commonly accepted threshold of 0.70, thus indicating good overall internal reliability. Further analysis revealed the alpha coefficients for the six core variables as follows: incentive-based environmental regulation (0.877), coercive environmental regulation (0.908), ecological cognition (0.936), descriptive social norms (0.951), injunctive social norms (0.920), and willingness to participate in S-FLC (0.944). Each of these values exceed the 0.80 benchmark for high reliability, demonstrating that the internal consistency of each subscale is robust, the item design is stable and repeatable, and the questionnaire exhibits a high level of reliability.

Second, to determine whether the data were suitable for structural factor analysis, the KMO test and Bartlett's test of sphericity were conducted. The KMO value for the overall scale was 0.868, significantly higher than the commonly accepted threshold of 0.60. Bartlett's test returned a significance level of *p* = 0.000, indicating that there is sufficient correlation among variables to justify factor analysis. Further calculation of KMO values for each of the six latent variables showed that all coefficients exceeded 0.60, further supporting the feasibility of conducting factor analysis.

Moreover, VIF and tolerance statistics were evaluated using latent variable scores exported from AMOS. All VIF values were below 3 and tolerance values were above 0.20, confirming no harmful multicollinearity existed among predictors. Additionally, to verify the absence of substantial common-method bias, Harman's single-factor test was performed, and the first factor accounted for 30.484% of the variance, which is less than the 40% threshold, indicating that CMV does not pose a serious threat to the interpretation of the results. In summary, the sample data used in this study exhibit strong structural suitability and a solid theoretical foundation, providing robust support for subsequent structural equation modeling analysis.

### Discriminant validity analysis

Further analysis was conducted on the discriminant validity of the measurement model from three dimensions: standardized factor loadings, composite reliability (CR), and average variance extracted (AVE). First, as shown in Table 2, the standardized factor loadings for all measurement items were above 0.60, indicating that each indicator adequately reflects its corresponding latent construct and that the model has strong structural validity. Second, the CR values for all latent variables exceeded the commonly accepted threshold of 0.70, suggesting that the scale demonstrates acceptable to excellent internal consistency and reliability. Third, the AVE values for all latent variables were greater than 0.50, indicating strong convergent validity, as each variable accounts for a substantial proportion of its indicator variance. For the discriminant validity test, the square root of the AVE for each latent construct was compared with the inter-construct correlations in the correlation matrix. The results show that the square root of each construct's AVE was greater than its correlations with any other construct, thus satisfying the Fornell-Larcker criterion. This confirms that the latent constructs in the model are empirically distinct from one another, despite theoretical correlations, and can be reliably distinguished at the statistical level.

**Table 2:** Path coefficient estimates and reliability/validity test results of measurement items.

**Construct**	**Item**	**Parameter significance estimate**				**Factor loading**	**Reliability**	**Composite reliability**	**Convergent validity**
		**Sth**.	**S.E**.	***t*** **-value**	***p***	**Sth**	**SMC**	**CR**	**AVE**
Incentive-based Environmental regulation	GR4	1.000				0.656	0.430	0.853	0.598
	GR3	1.055	0.055	19.172	^***^	0.646	0.417		
	GR2	1.274	0.086	14.779	^***^	0.883	0.780		
	GR1	1.321	0.091	14.463	^***^	0.874	0.764		
Coercive environmental regulation	BR4	1.000				0.875	0.766	0.910	0.718
	BR3	0.745	0.041	18.374	^***^	0.740	0.548		
	BR2	0.896	0.039	23.203	^***^	0.864	0.746		
	BR1	1.036	0.041	25.069	^***^	0.901	0.812		
Ecological cognition	EC1	1.000				0.893	0.797	0.921	0.702
	EC2	1.049	0.034	30.577	^***^	0.934	0.872		
	EC3	0.876	0.040	21.766	^***^	0.806	0.650		
	EC4	0.960	0.041	23.389	^***^	0.837	0.701		
	EC5	0.782	0.045	17.220	^***^	0.700	0.490		
Descriptive norms	DS3	1.036	0.036	29.028	^***^	0.898	0.806	0.937	0.831
	DS2	1.014	0.033	30.483	^***^	0.917	0.841		
	DS1	1.000				0.920	0.846		
Injunctive norms	IS3	1.013	0.033	31.131	^***^	0.903	0.815	0.952	0.870
	IS2	1.028	0.027	38.360	^***^	0.975	0.951		
	IS1	1.000				0.918	0.843		
Willingness to participate S-FLC	PW1	1.000				0.716	0.513	0.940	0.758
	PW2	1.366	0.071	19.331	^***^	0.918	0.843		
	PW3	1.349	0.070	19.402	^***^	0.925	0.856		
	PW4	1.472	0.078	18.906	^***^	0.899	0.808		
	PW5	1.399	0.076	18.457	^***^	0.878	0.771		

S-FLC, Sustainable Firecracker–Lion Dance Customs; ^***^p < 0.001.

To further ensure the validity of comparing local residents and tourists, multi-group measurement invariance testing supported configural, metric, and structural covariance equivalence (ΔCFI ≤ 0.010), indicating that the measurement properties are stable across groups. Additionally, a chi-square difference test strongly favored a two-factor structure of incentive-based vs. coercive-based regulation over a one-factor alternative (Δχ^2^ = 545.47, Δdf = 1, *p* < 0.001), confirming that these constructs are empirically distinct.

## Results

### Main effect testing

This study constructed a SEM to test the proposed hypotheses, using farmers' willingness to participate in the S-FLC as the dependent variable, environmental regulation as the independent variable, and social norms and ecological cognition as mediating variables. The model fit was evaluated using standard fit indices: the χ^2^/df value between 1 and 3 is considered acceptable; the CFI greater than 0.90 indicates an acceptable model fit; and the RMSEA below 0.08 indicates good model fit. In this study, all fit indices fall within the recommended thresholds, suggesting that the overall model fit is satisfactory. However, further examination of the initial measurement model indicates a suboptimal level of model-data compatibility (χ^2^/df = 3.975, RMSEA = 0.084, GFI = 0.820, NFI = 0.883). Based on these results, model refinement was necessary. Following standard procedures, items with sub-threshold standardized factor loadings were removed. Model modification was carried out based on standardized factor loadings and modification indices, and covariance relationships were added between error terms e1 and e2, and between e25 and e26, to improve model fit. The process was iterated until an optimal model fit was achieved. Following the main effect path estimation of the SEM, this study further conducted statistical testing and significance analysis on the path coefficients among the core variables. As shown in Table 3, incentive-based environmental regulation has a significant positive effect on farmers' willingness to participate in the S-FLC (β = 0.229, *p* < 0.001), indicating that under conditions where the government promotes environmentally friendly folk practices through education and incentive policies, farmers are more inclined to participate. Thus, Hypothesis H1a is supported. In contrast, the path coefficient between coercive environmental regulation and participation willingness is 0.059. Although the direction is positive, it does not meet the 1% significance level test (*p* > 0.01), indicating that coercive regulation may still provide some behavioral guidance but perhaps with limited strength under the current implementation context. Hypothesis H1b is not supported. Additionally, further analysis shows that both types of social norm variables have statistically significant positive effects on willingness to participate. Specifically, the standardized path coefficient of descriptive social norms is 0.167 (*p* < 0.01), and that of injunctive social norms is 0.238 (*p* < 0.001), indicating that whether through behavioral cues from the surrounding community (i.e., “what others do”) or through normative judgments based on social values and moral expectations (i.e., “what people think one should do”), social norms significantly enhance farmers' motivation and behavioral inclination to participate in folk customs. Therefore, Hypotheses H2a and H2b are supported.

**Table 3:** Main effect analysis results.

**Pathway**	**Standardized coefficient**	**S.E**.	**C.R**.	***p*-value**	**Hypothesis result**
Incentive-based environmental regulation → willingness to participate S-FLC	0.229	0.061	3.346	^***^	H1a supported
Coercive environmental regulation → willingness to participate S-FLC	0.059	0.041	1.414	0.157	H1b not supported
Injunctive norms → willingness to participate S-FLC	0.238	0.041	4.466	^***^	H2a supported
Descriptive norms → willingness to participate S-FLC	0.167	0.046	3.176	^**^	H2b supported
Ecological cognition → willingness to participate S-FLC	−0.210	0.036	−4.109	^***^	H4 supported

^***^ *p* < 0.001, ^**^ *p* < 0.01; S-FLC, Sustainable Firecracker–Lion Dance Customs.

Finally, ecological cognition shows a significant negative association with willingness to participate (β = −0.210, *p* < 0.001). This indicates that although the firecracker-lion dance has been environmentally improved under regulatory guidance, individuals with higher ecological awareness are still sensitive to remaining environmental risks, leading to a more cautious or reserved attitude toward participation. Therefore, Hypothesis H4 is supported. In summary, incentive-based regulatory tools and social norms play a crucial role in enhancing farmers' willingness to participate in traditional folk customs. In contrast, ecological cognition imposes significant behavioral constraints from a risk perception perspective, while conventional coercive regulatory measures demonstrate relatively limited effectiveness in the context of rural folk practices.

### Mediation effect analysis

To further explore how environmental regulation influences farmers' willingness to participate in S-FLC through social norms and ecological cognition, this study constructed a SEM incorporating dual mediating variables. Incentive-based environmental regulation and coercive environmental regulation were set as independent variables; social norms (including both descriptive and injunctive norms) and ecological cognition were introduced as mediators; and farmers' willingness to participate in S-FLC was treated as the dependent variable. Path analysis was conducted using AMOS 26.0, and the mediating effects were tested using the bootstrap method recommended by Shrout and Bolger (2002). Specifically, the bootstrap procedure was performed with 5,000 resamples, and the confidence interval was set at 95%. If the upper and lower bounds of the confidence interval do not include zero, the mediating effect is considered statistically significant (see Table 4).

**Table 4:** Mediation effect analysis results.

**Mediated pathway**	**Standardized indirect effect**	**S.E**.	**LLCI**	**ULCI**	**Hypothesis result**
Incentive-based environmental regulation → descriptive social norms → willingness to participate S-FLC	0.016	0.011	0.001	0.041	H3a supported
Incentive-based environmental regulation → injunctive social norms → willingness to participate S-FLC	0.035	0.018	0.004	0.075	H3c supported
Incentive-based environmental regulation → ecological cognition → willingness to participate S-FLC	0.002	0.012	−0.023	0.024	H5a not supported
Coercive environmental regulation → descriptive social norms → willingness to participate S-FLC	0.007	0.010	−0.010	0.029	H3b not supported
Coercive environmental regulation → injunctive social norms → willingness to participate S-FLC	0.029	0.016	0.000	0.063	H3d supported
Coercive environmental regulation → ecological cognition → willingness to participate S-FLC	−0.029	0.016	−0.063	−0.003	H5b supported

S-FLC, Sustainable Firecracker–Lion Dance Customs.

First, the analysis of the mediation pathways shows that both descriptive social norms (β = 0.016, *LLCI* = 0.001, *ULCI* = 0.041) and injunctive social norms (β = 0.035, *LLCI* = 0.004, *ULCI* = 0.075) exhibit significant partial mediation effects between incentive-based environmental regulation and willingness to participate in S-FLC. This suggests that within incentive-oriented regulatory frameworks, group behavioral demonstration and value-based social guidance act as effective behavioral bridges influencing participation in folk customs. However, the mediation effect of ecological cognition between incentive environmental regulation and willingness to participate S-FLC is not statistically significant, indicating that in policy contexts dominated by soft regulatory tools, ecological knowledge and environmental awareness have yet to form an effective explanatory pathway for behavior change. Additionally, when coercive environmental regulation serves as the independent variable, the total effect on participation willingness is no significant (β = 0.059, *p* = 0.157), both injunctive social norms (β= 0.029, *LLCI* = 0.002, *ULCI* = 0.063), and ecological cognition (β =-0.029, *LLCI* =-0.063, *ULCI* =-0.003), show significant indirect effects, indicating that much of the influence of coercive regulation is relayed through these mediating mechanisms rather than through a direct pathway.

This finding suggests that under coercive regulatory conditions, farmers are more likely to change their behavior by internalizing regulatory logic and aligning with existing social value structures, rather than directly responding to coercive policies. In summary, incentive-based regulation encourages farmers' participation in S-FLC by activating the demonstrative function of social norms, while coercive regulation impacts behavioral intentions through a dual pathway that involves social discipline and ecological risk perception. The differentiated mediation pathways reveal the unique behavioral incentive mechanisms associated with various regulatory tools in rural cultural contexts, offering empirical support at the behavioral mechanism level for the future design of environmental policies. A Monte-Carlo power analysis (1,000 replications; N = 423) was conducted by fixing all structural coefficients at a conservative effect size of β = 0.05. This generated very small indirect effects (≈0.0025) and correspondingly low statistical power ( ≤ 0.003), suggesting that nonsignificant mediation paths likely reflect trivial effects rather than inadequate sample size. Notably, several indirect effects still reached significance in the empirical model, indicating that these relationships are robust and theoretically meaningful.

## Discussion and conclusion

### Discussion

Based on 423 valid survey responses collected in Deqing County, Guangdong Province, this study employed SEM and bootstrap mediation testing to examine the influence pathways of environmental regulation on farmers' willingness to participate in S-FLC, as well as the underlying psycho-social mechanisms, particularly focusing on the dual mediating effects of social norms and ecological cognition. The findings not only confirm the varying impacts of different regulatory approaches but also reveal the intricate and often conflicting interactions between policy instruments and cultural practices.

First, incentive-based environmental regulation has been shown to significantly enhance farmers' willingness to engage in the S-FLC. This finding indicates that regulatory strategies that employ a reward-based, motivational approach, while respecting and accommodating the core values of traditional culture and promoting its transition toward greener, lower-pollution practices, can foster broader participation. This could lead to mutually beneficial outcomes for both ecological governance and cultural preservation (Xiong et al., 2022). As Tietenberg (1998) has noted, market-based regulatory tools can achieve environmental objectives through flexible implementation without undermining the agency or engagement of individual actors. In contrast, coercive environmental regulation shows a positive yet statistically non-significant effect on participation willingness (β = 0.059, *p* > 0.05), suggesting that coercive measures alone may not be sufficient to meaningfully shift participation behavior in the context of culturally loaded practices. A possible explanation is that coercive regulation mainly relies on compliance pressure rather than internal motivation. When formal rules are not fully aligned with farmers' cultural expectations, their behavioral change may be incremental rather than immediate (Gelfand et al., 2020). The role of social norms is further highlighted in this study. Findings reveal that both descriptive norms and injunctive norms significantly enhance farmers' willingness to participate in S-FLC. In the context of highly interpersonal rural social structures, neighborhood behavioral modeling and collective moral expectations function as “soft rules” that regulate everyday behavior (Bicchieri et al., 2022). To some extent, these norms form an informal embedded support system for environmental policies. Even when the policies themselves lack persuasive appeal, social norms can internalize them as community-level consensus, thereby indirectly enhancing regulatory effectiveness (Li and Wu, 2021; Zhang et al., 2021).

More importantly, this study finds that injunctive norms fully mediate the relationship between coercive environmental regulation and participation willingness. This elucidates a pivotal insight: only when policy content is articulated in moral terms and acknowledged by the community as an imperative can stringent regulation be converted into behavioral motivation (Cui and Liu, 2022). This points to a deeper socio-psychological mechanism—farmers' compliance with policy is not merely driven by fear of punishment, but rather by a normative internal drive to align with the prevailing system of social evaluation. In addition, ecological cognition, serving as a cognitive mediator, follows a negative path of influence. Farmers with stronger ecological awareness are more likely to recognize the environmental risks associated with the firecracker-lion dance, such as air pollution and noise disturbance. This awareness in turn suppresses their behavioral tendency to participate in environmentally harmful traditional practices. This finding extends the applicability of ecological cognition beyond productive behaviors (e.g., green farming or resource use), suggesting that cognitive regulation also plays an important role in behavior domains intertwined with cultural and emotional significance.

However, ecological cognition did not demonstrate a significant mediating effect in the context of incentive-based regulation, but it did reveal a notable negative mediating effect under coercive regulation. This observation points to two potential psychological mechanisms at play. First, the high-pressure environment created by administrative regulation may trigger risk associations, thus heightening individuals' awareness of ecological consequences. Second, the policy transmission process may lead to a cultural distancing effect, resulting in a conflict of “cultural–ecological dual identity.” In this scenario, individuals find themselves grappling with internal tensions between their traditional cultural identity and their sense of ecological responsibility. In conclusion, this study finds that environmental regulation does not operate uniformly across all contexts; its effectiveness is highly contingent on the “soft embeddedness” of social norms and the “rational coordination” of ecological cognition. Policymakers should pay greater attention to the holistic nature of cultural ecologies and the complexity of behavioral mechanisms, avoiding the tendency to treat regulation as a linear cause-effect solution. Instead, policy design should consider multi-level, multidimensional linkages across social, cultural, and cognitive domains.

Despite systematically revealing the mechanisms through which environmental regulation, social norms, and ecological cognition influence farmers' willingness to participate in S-FLC and proposing a behavior model applicable to contexts of cultural–ecological tension, this study still has several limitations that warrant further investigation and refinement in future research. First, although the causal structure proposed in this study is theoretically grounded and empirically supported, the cross-sectional and self-reported nature of the data still limits causal inference. For example, individuals with a stronger willingness to participate may reinterpret regulatory information in line with their existing attitudes. Therefore, future research may adopt longitudinal or experimental designs to verify the causal chain and strengthen causal validity. Moreover, participants were sampled from the town center and multiple villages; however, uneven sample sizes across villages prevented the use of a multilevel SEM design. Future research may benefit from balanced hierarchical sampling and multilevel modeling to better account for village-level clustering effects. Furthermore, although all variables were collected from the same questionnaire, the risk of CMV influencing the mediation results is minimal. According to Lindell and Whitney (2001), CMV can't produce theoretically coherent sequential mediation patterns across distinct constructs. In this study, multiple mediators with confirmed discriminant validity jointly generate indirect paths that are statistically aligned with theoretical expectations (Lindell and Whitney, 2001), which means that CMV is unlikely to invalidate the substantive mediation mechanisms identified in this study.

As environmental regulations become more stringent, traditional festivals associated with pollution risks are facing increasing restrictions. Achieving a dynamic balance between environmental protection and cultural continuity, while preventing cultural erosion and avoiding backlash against policies, has become a critical and pressing challenge. Although the firecracker-lion dance is a highly spectacular yet hazardous ritual, the key findings can be applied to a broader context. The positive impact of incentive-based regulation is likely relevant to a variety of community customs, as external rewards may enhance the willingness to uphold cultural practices sustainably. Similarly, the significant influence of descriptive and injunctive norms suggests the established role of collective identity and social approval within rural communities. These mechanisms are expected to occur in low-intensity rituals such as paper-offering and festival songs.

### Conclusion and policy implications

Using the firecracker-lion dance tradition in Deqing, Guangdong, as a case study, this research empirically examined how environmental regulation influences farmers' willingness to participate in S-FLC, particularly focusing on the mediating roles of social norms and ecological cognition. The findings indicate that incentive-based environmental regulation significantly increases farmers' willingness to participate, while coercive environmental regulation has a positive effect that is not statistically significant. This suggests that the impact of mandatory policy instruments may be comparatively weaker in culturally embedded contexts. Moreover, social norms, acting as culturally embedded drivers of behavior, play a critical role in guiding farmers' decision-making, with injunctive norms particularly enhancing the effectiveness of policy measures. In contrast, ecological cognition appears to significantly suppress participation willingness, implying that ecological values may conflict with certain cultural practices. Furthermore, the mediation analysis shows that both injunctive norms and ecological cognition mediate the relationship between coercive regulation and behavioral intention. This emphasizes that cultural compatibility and value alignment are essential mechanisms for achieving policy effectiveness in the governance of traditional cultural activities.

Based on the above research findings, the following policy recommendations are proposed to provide practical pathways for the coordinated advancement of traditional cultural preservation and ecological environmental governance:

(1) Take incentive-based environmental regulation as the core to construct a culturally adaptive environmental protection framework. The government can guide farmers to adopt more sustainable forms of expression (such as electronic firecrackers or simulated fireworks) while retaining traditional symbols and ritual structures by providing environmental subsidies and supporting green transformation technologies (Wang et al., 2024). In the process of policy formulation, the situational and ritual nature of folk culture should be respected. A “policy-community” consultation mechanism may be explored. For example, through villagers' representative assemblies, to participate in setting standards for timing, location, and graded control of firecracker use.

(2) Strengthen the governance effectiveness of social norms and promote the integration of “soft regulation” and “group consensus.” The network structure and moral rules of rural acquaintance societies provide natural soil for the operation of social norms (Li B. et al., 2022). It is recommended to incorporate environmental protection behavior into village regulations and agreements, enhancing the moral constraint function of injunctive norms. By selecting exemplary models such as “Green Folk Practice Families,” descriptive norms can be shaped to create positive behavioral samples and exert exemplary effects. New media platforms should be fully utilized to disseminate typical cases of green folk customs and construct a “visualized collective consensus,” expanding the spatial and temporal coverage of normative influence.

(3) Promote the coordinated enhancement of ecological cognition and cultural identity, and deepen participatory educational approaches. The enhancement of ecological cognition should not be built on the basis of cultural negation or suppression but should be embedded in the ecological wisdom within the culture itself. It is recommended to implement a “participatory cognition enhancement” strategy by organizing farmers to participate in ecological perception activities such as air quality monitoring and water testing to improve their intuitive understanding of pollution consequences. Meanwhile, invite intangible cultural heritage inheritors to explain the ecological values contained in folk customs, such as nature worship and seasonal logic, and promote the modern transformation of traditional knowledge.

(4) Optimize the implementation mechanism of coercive regulation and embed social evaluation to improve policy acceptance. When implementing necessary restrictive measures, introduce a mechanism of “soft enforcement + social evaluation.” For addressing violations, consider utilizing neighborhood mediation or collective deliberation mechanisms to prevent resistance and backlash resulting from administrative enforcement.

## Data Availability

The datasets presented in this article are not readily available due to privacy and ethical restrictions, but it can be provided by the authors upon reasonable request for academic research purposes. Requests to access the datasets should be directed to Jiaxiao Feng, 1875102787@qq.com.

## Appendix A

**Table A1:** Measurement items.

**Construct**	**Code**	**Item**
Willingness to participate (PW)	PW1	I am familiar with the “Firecracker-Lion Dance” activity.
	PW2	I enjoy the “Firecracker-Lion Dance” activity when it is performed in an environmentally friendly and regulated manner.
	PW3	I support continuing this traditional practice as long as it complies with environmental protection requirements.
	PW4	I am very willing to participate in the “Firecracker-Lion Dance” activity when it is performed in an environmentally friendly and regulated manner.
	PW5	I will continue participating in the “Firecracker-Lion Dance” activity next year.
Incentive-based environmental regulation (GR)	GR1	The government has made strong efforts to educate the public about policies restricting firecracker use.
	GR2	The public education on firecracker restriction policies has been effective.
	GR3	I agree that rewards should be given to those who strictly comply with firecracker prohibition policies.
	GR4	I agree that those who strictly comply with firecracker prohibition policies should be publicly recognized or praised.
Coercive environmental regulation (BR)	BR1	I agree that fines should be imposed on illegal firecracker use.
	BR2	I agree that those who illegally set off firecrackers should receive criticism and education.
	BR3	Government agencies have effectively monitored and regulated firecracker use.
	BR4	I agree that administrative punishment should be applied to illegal firecracker use.
Descriptive social norms (DS)	DS1	My relatives set off firecrackers during festivals or celebrations.
	DS2	My neighbors set off firecrackers during festivals or celebrations.
	DS3	My friends set off firecrackers during festivals or celebrations.
Injunctive social norms (IS)	IS1	My relatives think that firecrackers should be used during the “Firecracker-Lion Dance” activity.
	IS2	My neighbors think that firecrackers should be used during the “Firecracker-Lion Dance” activity.
	IS3	My friends think that firecrackers should be used during the “Firecracker-Lion Dance” activity.
Ecological cognition (EC)	EC1	Setting off firecrackers during the “Firecracker-Lion Dance” activity pollutes the ecological environment.
	EC2	Setting off firecrackers during the “Firecracker-Lion Dance” activity deteriorates air quality.
	EC3	Setting off firecrackers during the “Firecracker-Lion Dance” activity causes noise pollution.
	EC4	Firecracker emissions from the “Firecracker-Lion Dance” activity cause long-lasting air pollution.
	EC5	I believe that reducing firecracker use is important for environmental protection.
